# Comparison of *F*-tests for Univariate and Multivariate Mixed-Effect Models in Genome-Wide Association Mapping

**DOI:** 10.3389/fgene.2019.00030

**Published:** 2019-02-04

**Authors:** Akio Onogi

**Affiliations:** ^1^Institute of Crop Science, National Agriculture and Food Research Organization, Tsukuba, Japan; ^2^Japan Science and Technology Agency PRESTO, Kawaguchi, Japan

**Keywords:** genome-wide association study, GWAS, multiple traits, quantitative traits, QTL mapping, multitask, linear mixed model

## Abstract

Genome-wide association mapping (GWA) has been widely applied to a variety of species to identify genomic regions responsible for quantitative traits. The use of multivariate information could enhance the detection power of GWA. Although mixed-effect models are frequently used for GWA, the utility of *F*-tests for multivariate mixed-effect models is not well-recognized. Thus, we compared the *F*-tests for univariate and multivariate mixed-effect models with simulations. The superiority of the multivariate *F*-test over the univariate test varied depending on three parameters: phenotypic correlation between variates (*r*), relative size of quantitative trait locus effects between variates (*a*_d_), and missing proportion of phenotypic records (*m*_prop_). Simulation results showed that, when *m*_prop_ was low, the multivariate *F*-test outperformed the univariate test as *r* and *a*_d_ differ, and as *m*_prop_ increased, the multivariate *F*-test outperformed as *a*_d_ increased. These observations were consistent with results of the analytical evaluation of the *F*-value. When *m*_prop_ was at the maximum, i.e., when no individual had phenotypic values for multiple variates, as in the case of meta-analysis, the multivariate *F*-test gained more detection power as *a*_d_ increased. Although using multivariate information in mixed-effect model contexts did not always ensure more detection power than with univariate tests, the multivariate *F*-test will be a method applied when multivariate data are available because it does not show inflation of signals and could lead to new findings.

## Introduction

Genome-wide association mapping (GWA) has been widely applied to humans, animals, and plants to identify genomic regions responsible for quantitative traits, which has been made feasible by decreases in the cost and time required to obtain genome-wide single-nucleotide polymorphisms (SNPs) and sequences. Whereas various statistical methods have been proposed for GWA, particularly in recent animal and plant studies, mixed-effect models are often used to correct population stratification (e.g., Zhao et al., 2011; Sahana et al., 2014; Yano et al., 2016; Frischknecht et al., 2017) inspired by Yu et al. (2006). The detection power of GWA primarily depends on the sample size, which is common for statistical methods. Thus, when few samples (genotypes) are available, as often seen in plant studies (e.g., Zhao et al., 2011; Yano et al., 2016; Minamikawa et al., 2017), GWA may fail to detect any responsible regions or, even if it does, only a few regions are found.

In contrast, biological data are usually multivariate. For example, phenotypes are often measured for multiple traits. Phenotypes may be measured on multiple occasions or in different locations and/or by multiple individual using a variety of methods. For plants, multi-year and/or multi-environment evaluation is often conducted to understand the complex reactions of plants to environmental stimuli, which can be observed as genotype-by-environment interactions. Moreover, meta-analysis or phenotypes belonging to different groups of individuals, for example, sex, age, or geographical populations, can be considered as multivariate with the phenotypic record of each sample consisting of only a single variate.

Thus, the use of multivariate information to enhance the detection power of GWA is straightforward. Indeed, various methods for multivariate GWA or quantitative trait locus (QTL) mapping have been proposed (Piepho, 2005; Banerjee et al., 2008; Ferreira and Purcell, 2009; Kim and Xing, 2009; O'Reilly et al., 2012; Zhang et al., 2014; Guo et al., 2015; Ray et al., 2016; Wang et al., 2016; Cheng et al., 2017), and these methods have been compared (Galesloot et al., 2014). However, these methods do not include polygenic effects, essential parts of mixed-effect models, which are effective for adjustment of population structure. Although GWA based on multivariate mixed-effect models has also been studied, the focus has been on computational efficiency (Zhou and Stephens, 2014; Furlotte and Eskin, 2015; Joo et al., 2016), and not on the properties of statistical tests. Simulation analysis for statistical tests based on multivariate mixed-effect models conducted to date remains insufficient in terms of the ranges of scenarios; relative QTL effect sizes among variates, phenotypic correlation, and the missing proportion of phenotypes are not fully considered (e.g., Korte et al., 2012; Zhou and Stephens, 2014).

In the present study, we formulated the *F*-test for multivariate mixed-effect models of general form; both empirical and analytical evaluations were performed to elucidate the properties of the *F*-tests for multivariate mixed-effect models. The *F*-test (or the Wald-type test) is usually used for association analysis based on univariate mixed-effect models (e.g., R package, rrBLUP, Endelman, 2011). The multivariate *F*-test simultaneously tests the effect of SNPs on multiple variates. Thus, the purpose of the multivariate *F*-test introduced here is to identify genomic regions that are common to multiple variates and cannot be detected by the univariate test for each variate.

## Materials and Methods

### Univariate *F*-test in GWA

GWA is often conducted assuming the following univariate mixed-effect model,

$$\begin{array}{l} \text{Y}=\text{X}\text{B}+\text{Z}\text{u}+\text{e}, \end{array}$$

where **Y** is the vector of response variables (e.g., phenotypic records), **X** is the covariate matrix, including intercepts, genotypes of the SNP to be tested, and principal components for adjustment of population structure, **B** is the fixed effects of the covariates, **Z** is the design matrix, **u** is the polygenic effects, and **e** is the residuals. **u** and **e** follow multivariate normal distributions,

$$\begin{array}{l} u\sim \text{MVN}\left(\begin{array}{c} 0,A\sigma_{u}^{2} \end{array}\right), \end{array}$$

where **A** is the genomic relationship matrix defined by genome-wide SNPs (e.g., VanRaden, 2008), and $\sigma_{u}^{2}$ is the additive genetic variance explained by SNPs and

$$\begin{array}{l} e\sim \text{MVN}\left(\begin{array}{c} 0,I\sigma_{e}^{2} \end{array}\right), \end{array}$$

where $\sigma_{e}^{2}$ is the residual variance. The significance of **B** can be assessed by the *F*-test (Henderson, 1984; Kennedy et al., 1992). The *F*-statistic is

(1) $$\begin{array}{r} F=\frac{{\left({\text{H}}^{\prime }\hat{\text{B}}\right)}^{\prime }{\left[{\text{H}}^{\prime }{\left({\text{X}}^{\prime }{\text{V}}^{-1}\text{X}\right)}^{-1}\text{H}\right]}^{-1}\left({\text{H}}^{\prime }\hat{\text{B}}\right)}{f\hat{\sigma^{2}}}. \end{array}$$

Here, **V** is the phenotypic covariance, i.e., $V=ZAZ^{\prime }\sigma_{u}^{2}+I\sigma_{e}^{2}$, and $\hat{\text{B}}={\left({\text{X}}^{\prime }{\text{V}}^{-1}\text{X}\right)}^{-1}{\text{X}}^{\prime }{\text{V}}^{-1}\text{Y}$. *f* is the number of fixed effects to be tested, and

$$\begin{array}{l} \hat{\sigma^{2}}=\frac{{\left(\text{Y}-\text{X}\hat{\text{B}}\right)}^{\prime }{\text{V}}^{-1}\left(\text{Y}-\text{X}\hat{\text{B}}\right)}{\text{n}-\text{p}}=1, \end{array}$$

where *n* is the number of individuals and *p* is the number of fixed effects. **H** is a matrix to indicate which effects in $\hat{\text{B}}$ are tested. For example, when *p* is five and the second effect is tested, **H**′ = [0 1 0 0 0]. Here we assume that when the fixed effect of interest is 0, the *F*-statistic follows an *F* distribution with the numerator degrees of freedom being *f* and the denominator degrees of freedom being *n* − *p*. We refer to this test as the univariate *F*-test. However, note that this assumption ignores the reduction of the denominator degrees of freedom owing to estimation of variance components. Because of this reduction, the *F*-test introduced here can underestimate (inflate) *p* (−log10P) values. Nevertheless, we propose that the methods and results presented here are useful in practice because *p*-values obtained for negative SNPs (i.e., SNPs that were unlinked to QTLs) were not underestimated in simulations presented later, and thus the reduction of degrees of freedom would not affect GWA given the number of samples usually used in GWA. This issue is revisited in the Results and Discussion.

### Multivariate *F*-test

A multivariate mixed model can be written as

$$\begin{array}{l} {\text{Y}}_{\text{m}}={\text{X}}_{\text{m}}{\text{B}}_{\text{m}}+{\text{Z}}_{\text{m}}{\text{u}}_{\text{m}}+{\text{e}}_{\text{m}}. \end{array}$$

Here, **Y**_m_ is the vector of response variables of length *n* × *d*, where *d* is the number of variates (e.g., traits or experiment years). **X**_m_ is the matrix of covariates, including the intercepts, SNP genotypes, and so on. For example, when *d* is two,

$$\begin{array}{l} {\text{X}}_{\text{m}}=\left[\begin{array}{c} \text{X}\;0\\ 0\;\text{X} \end{array}\right]. \end{array}$$

**B**_m_ is the vector of fixed effects of length *p* × *d* where *p* is the number of fixed effects for each variate. **Z**_m_ is the design matrix, which for the example of *d* = 2, takes the following form,

$$\begin{array}{l} {\text{Z}}_{\text{m}}=\left[\begin{array}{c} \text{Z}\;0\\ 0\;\text{Z} \end{array}\right]. \end{array}$$

**u**_m_ and **e**_m_ are the polygenic effects and residuals, respectively, and

$$\begin{array}{l} {\text{u}}_{\text{m}}\sim \text{MVN}\left(0,\;\Sigma_{\text{u}}^{2}⊗\text{A}\right), \end{array}$$

where ⊗ indicates the Kronecker product and $\Sigma_{\text{u}}^{2}$ is the *d* × *d* genetic covariance matrix, and

$$\begin{array}{l} {\text{e}}_{\text{m}}\sim \text{MVN}\left(0,\;\Sigma_{\text{e}}^{2}⊗\text{I}\right), \end{array}$$

where $\Sigma_{\text{e}}^{2}$ is the *d* × *d* residual covariance matrix.

In the *F*-test introduced here for this multivariate mixed-effect model, SNP effects on all of the variates are tested simultaneously. The *F*-statistic in the multivariate case is

(2) $$F=\frac{{\left(H^{\prime }\hat{B_{m}}\right)}^{\prime }{\left[H^{\prime }{\left({\text{X}}_{\text{m}}^{\prime }{\text{V}}_{\text{m}}^{-1}{\text{X}}_{\text{m}}\right)}^{-1}H\right]}^{-1}\left(H^{\prime }\hat{B_{m}}\right)}{f\hat{\sigma_{m}^{2}}}.$$

Here, $V_{m}=\Sigma_{u}^{2}⊗ZAZ^{\prime }+\Sigma_{e}^{2}⊗I$, $\hat{{\text{B}}_{\text{m}}}={\left({\text{X}}_{\text{m}}^{\prime }{\text{V}}_{\text{m}}^{-1}{\text{X}}_{\text{m}}\right)}^{-1}{\text{X}}_{\text{m}}^{\prime }{\text{V}}_{\text{m}}^{-1}{\text{Y}}_{\text{m}}$, and

$$\begin{array}{l} \hat{\sigma_{m}^{2}}=\frac{{\left({\text{Y}}_{\text{m}}-{\text{X}}_{\text{m}}\hat{{\text{B}}_{\text{m}}}\right)}^{\prime }{\text{V}}_{\text{m}}^{-1}\left({\text{Y}}_{\text{m}}-{\text{X}}_{\text{m}}\hat{{\text{B}}_{\text{m}}}\right)}{n\times d-p}=1. \end{array}$$

We assume that when the fixed effect of interest is 0, the *F*-statistic follows an *F* distribution with the numerator degrees of freedom being *f* and the denominator degrees of freedom being *n* × *d* − *p*. When **Y**_m_ includes missing records, the dimensions of **Y**_m_, **X**_m_, and **V**_m_ and the denominator of $\hat{\sigma_{m}^{2}}$ decrease accordingly, excluding the missing records from the model. In this multivariate setting, **H** is constructed as follows. For example, when *d* is two (variates 1 and 2) and ${\text{B}}_{\text{m}}^{\prime }=\left[\mu_{1}\;B_{1}\;\mu_{2}\;B_{2}\right]$, where μ_1_ and μ_2_ are the intercepts and *B*_1_ and *B*_2_ are the effects of a SNP, **H** becomes

$$\begin{array}{l} {\text{H}}^{{}^{\prime }}=\left[\begin{array}{cccc} 0 & 1 & 0 & 0\\ 0 & 0 & 0 & 1 \end{array}\right]. \end{array}$$

We refer to this test as the multivariate *F*-test.

Korte et al. (2012) used the *F*-test for bivariate mixed-effect models, and GEMMA provides the Wald, likelihood ratio, and score tests (Zhou and Stephens, 2014). The *F*-test is asymptotically equivalent to the Wald test, and the likelihood ratio test is equivalent to the Wald test when the parameters except for the one to be tested are equal in the null and alternative models. Furlotte and Eskin (2015) and Joo et al. (2016) also provide the multivariate *F*-tests based on transformed matrices for high computational efficiency.

### Simulation

#### Marker Genotypes

Genome-wide markers were generated using a coalescent simulator, Genome (Liang et al., 2007). We assumed a diploid selfing species because small sample sizes are often seen in crop studies (e.g., 176 rice varieties in the study by Yano et al., 2016). The number of chromosomes was five, and it was assumed that there were 2,000 SNPs on each chromosome. There were 200 individuals. The parameters of Genome used for the simulation were as follows: “-pop 1 200 -N 1000 -c 1 -pieces 2000 -s 2000 -rec 0.0002.” A typical distribution of linkage disequilibrium among SNPs is shown in Figure S1. SNP genotypes were generated 100 times with the simulator. We pruned SNPs with a minor allele frequency < 0.02, which resulted in the average number of SNPs being 6390 (± 82). Using these genotype data, 100 data sets were generated for each scenario described below.

#### QTL Effects and Phenotypes

We selected four SNPs per chromosome randomly as QTLs (i.e., a total of 20 QTLs). Among the QTLs, one QTL was randomly selected as a “target QTL,” and the remaining ones were used as “background QTLs.” For the background QTLs, the additive genetic effects were simulated using a multivariate normal distribution,

$$\begin{array}{l} {\text{B}}_{\text{m},\text{b}QT\text{L}}\sim \text{MVN}\left(0,\;\Sigma \right) \end{array}$$

where **B**_m,bQTL_ is a vector of background QTL effects with length *d* (number of variates), and ***Σ*** is a *d* by *d* correlation matrix represented with a single correlation parameter *r*, i.e.,

$$\begin{array}{l} \Sigma =\left[\begin{array}{c} 1\;\cdots \;r\\ \vdots \;\ddots \;\vdots \\ r\;\cdots \;1 \end{array}\right]. \end{array}$$

For the target QTL, the QTL effect on the first variate was generated from the standard normal distribution,

$$\begin{array}{l} B_{\text{tQTL},1}\sim N\left(0,\;1\right), \end{array}$$

and then the effect on the *j*th variate (*d* ≥ *j* > 1) was determined as

$$\begin{array}{l} B_{\text{tQTL},\text{j}}=a_{j}B_{\text{tQTL},1}, \end{array}$$

where *a*_*j*_ is a real value between −1 and 1. We investigated the detection power of the multivariate *F*-test by assessing the power on the target QTLs while varying *a*_*j*_ and *r* values. Total genetic effects, **u**_m_, were generated by summing all of the products between the QTL effects and QTL genotypes. The residuals of individual *i* were generated using the same correlation matrix ***Σ***,

$$\begin{array}{l} {\text{e}}_{\text{m},\text{i}}\sim \text{MVN}\left(0,\;\text{S}\Sigma \text{S}\right). \end{array}$$

where **S** is a diagonal matrix whose elements were the standard deviations of the total genetic effects (**u**_m_). Thus, the heritability was 0.5 throughout the simulation. The phenotypic correlation was largely ***Σ***. Because, as illustrated in the previous section, the *F*-value is influenced by the phenotypic covariance (**V**) rather than the individual variance components ($\Sigma_{\text{u}}^{2}$ and $\Sigma_{\text{e}}^{2}$), we did not simulate situations where the genetic and residual correlations differ.

We set *d* to 2, 4, and 8. When *d* = 2, *r* was set to −0.95, −0.9, −0.8, …, 0.8, 0.9, and 0.95. When *d* = 4 and 8, *r* was 0, 0.4, 0.8, and 0.95. For example, when *d* = 4 and *r* is 0.8, ***Σ*** is

$$\begin{array}{l} \left[\begin{array}{cccc} 1 & 0.8 & 0.8 & 0.8\\ 0.8 & 1 & 0.8 & 0.8\\ 0.8 & 0.8 & 1 & 0.8\\ 0.8 & 0.8 & 0.8 & 1 \end{array}\right] \end{array}$$

When *d* = 2, *a*_d_ was −1, −0.95, −0.9, −0.8, …, 0.8, 0.9, 0.95, and 1. When *d* = 4 and 8, *a*_d_ was 0, 0.1, 0.2, …, 0.9, 0.95, and 1. Then, *a*_*j*_ (*d* > *j* >1) was determined as the intermediates between 1 and *a*_d_ with constant intervals. For example, when *d* = 4 and *a*_4_ = 0.4, *a*_2_ = 0.8 and *a*_3_ = 0.6, respectively.

We did not assess the detection power of *F*-tests using the background QTLs. The rationale for this is as follows. When we draw random variables from a multivariate normal distribution and represent the variables using *a*_d_, a wide range of *a*_d_ values are obtained. For example, when we draw random values from

$$\begin{array}{l} MVN\left(0,\;\left[\begin{array}{c} 1\;0.95\\ 0.95\;1 \end{array}\right]\right), \end{array}$$

Approximately 80% of variable pairs are represented with *a*_d_ < 0.9, and even 10% of pairs show *a*_d_ < 0. However, as we show later, *a*_d_ has an impact on the detection power of multivariate *F*-tests. Thus, using the background QTLs, the power of multivariate *F*-tests could not be assessed appropriately.

#### Missing Phenotypes

Missing phenotypic values were randomly generated, such that every individual had a phenotypic value for at least one variate. Thus, when *d* = 2, 4, and 8, the maximum missing proportions of phenotypic values were (2 × 200–200)/(2 × 200) = 0.5, (4 × 200–200)/(4 × 200) = 0.75, and (8 × 200–200)/(8 × 200) = 0.875, respectively (200 is the number of individuals). When *d* = 2, *m*_prop_ was set to 0, 0.125, 0.25, 0.375, and 0.5; when *d* = 4, *m*_prop_ was 0, 0.188, 0.375, 0.563, and 0.75; and when *d* = 8, *m*_prop_ was 0, 0.219, 0.438, 0.656, and 0.875. Note that when *m*_prop_ is the maximum value, every individual has a phenotypic value only for a single variate. When *m*_prop_ > 0, *r* was set to 0, 0.1, …, 0.8, 0.9, 0.95, and *a*_d_ was 0, 0.1, 0.2, …, 0.9, 0.95, and 1 when *d* = 2. When *d* = 4 and 8, *r* was 0, 0.4, 0.8, and 0.95, and *a*_d_ and *a*_*j*_ (*d* > *j* >1) were set as when *m*_prop_ = 0.

In the analysis of each scenario, we used SNPs that were not selected as QTLs to construct the genomic relationship matrix for the polygenic effects. Then, the effect of the target QTL was tested. Principal components were not added. Other than the QTL genotypes, only intercepts for each variate were added to the model as the fixed effects.

### Implementation

The univariate and multivariate *F*-tests were implemented using R (R Development Core Team, 2011). Variance components ($\sigma_{u}^{2}$, $\sigma_{e}^{2}$, $\Sigma_{\text{u}}^{2}$, and $\Sigma_{\text{e}}^{2}$) were estimated using remlf90 software (Misztal et al., 2002), which is based on an EM algorithm. The estimation of variance components was conducted using the null model, i.e., a model that did not include any SNP genotypes as fixed effects; then, *F*-tests were performed for each SNP using the estimated variance components. This approximation procedure was proposed in Kang et al. (2010) and Zhang et al. (2010). R scripts for the multivariate *F*-tests are available at https://github.com/Onogi/MultivariateFtest. Simulated data and analysis results are available upon request.

## Results and Discussion

We assessed the power of the multivariate *F*-test by comparing the *p*-values for the target QTLs obtained by the multivariate and univariate *F*-tests. Because the additive effect of the target QTL on the first variate was simulated to be greatest among the variates (see Materials and Methods), we compared the–log10*p*-values of the multivariate *F*-tests with those of the univariate tests for the first variate, which was expected to be highest among the variates.

**Figure 1:**
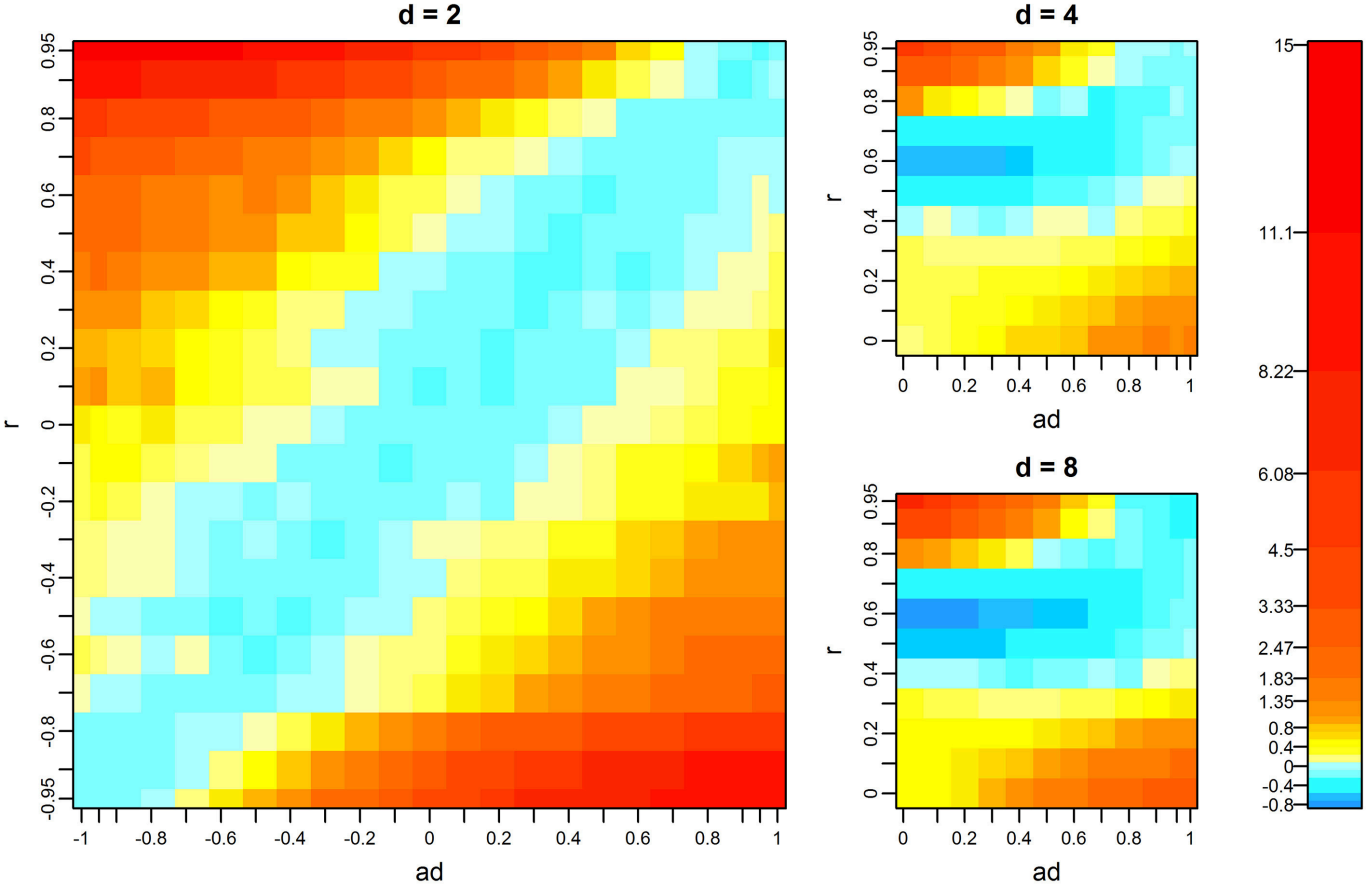
Mean difference of –log10*p* values between the multivariate and univariate *F*-tests. The *p*-values of the univariate *F*-test were obtained from the test on the first variate, which had the greatest QTL effect. Warm colors indicate that the multivariate *F*-test showed higher–log10*p*-values than the univariate test, and cold colors indicate that the univariate *F*-test showed higher values. *d, r*, and *a*_d_ denote the number of variates, phenotypic correlation, and the relative size of QTL effects between variates, respectively. Missing proportions of phenotypes are zero. For the results when *d* = 4 and *d* = 8, the mean differences of the scenarios that were not tested were interpolated using spline regression implemented in the mgcv R package.

**Figure 2:**
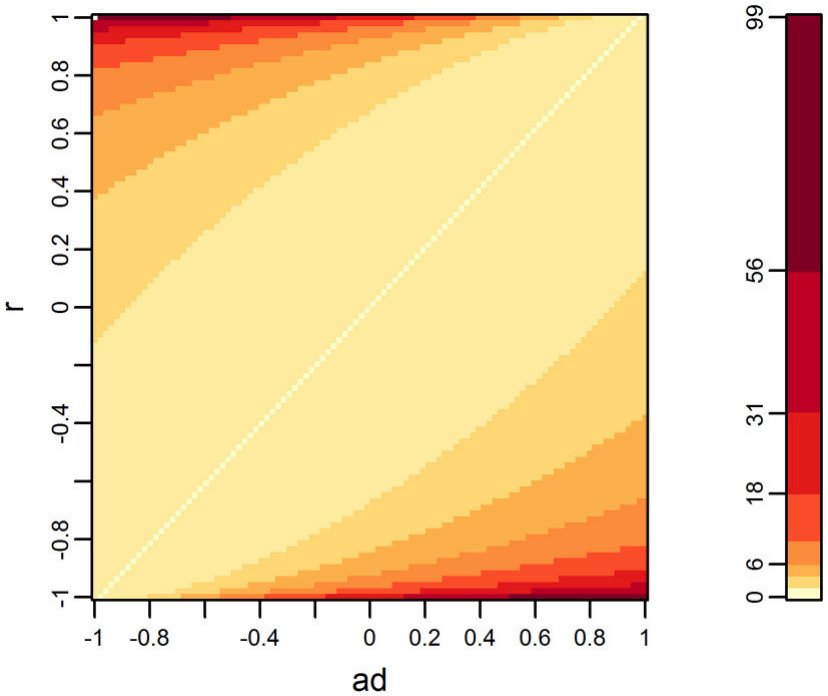
Distribution of *f* (*a*_2_, *r*) when the number of variates is two. *f* (*a*_2_, *r*) is a term appearing in equation (3), which is the numerator of the *F*-value of the multivariate *F*-test, making several assumptions for simplification (see the main text). Higher values are relevant with lower *p*-values.

When *d* = 2 and the data had no missing records, the multivariate *F*-test outperformed the univariate test as the differences between *a*_d_ and *r* became large (Figure 1). This tendency was also confirmed by analytical evaluation of the numerator of the *F*-value in the multivariate *F*-test (Equation 2). The numerator can be expressed as a function of *a*_d_ and *r* by simplifying model assumptions as follows. Suppose that *d* = 2, $V=\left[\begin{array}{cc} 1 & r\\ r & 1 \end{array}\right],\hat{\beta_{\text{tQTL},2}}=a_{2}\hat{\beta_{\text{tQTL},1}}$, and **A** = **I**. Then, the numerator of the *F*-value is

(3) $$\begin{array}{r} {\hat{\beta_{\text{tQTL},1}}}^{2}{\text{X}}^{\prime }\text{X}\frac{a_{2}^{2}-2a_{2}r+1}{1-r^{2}}={\hat{\beta_{\text{tQTL},1}}}^{2}{\text{X}}^{\prime }\text{X}f\left(a_{2},r\right) \end{array}$$

where **x** is the vector of the genotypes of the QTL. The heat map of *f* (*a*_2_, *r*), shown in Figure 2, is consistent with the heat map when *d* = 2, shown in Figure 1.

The results when *d* = 4 and 8 differ from that when *d* = 2, particularly when both *r* and *a*_d_ are near zero (Figure 1). This finding is probably because we determined *a*_*j*_ (*d* > *j* >1) as the intermediate between 1 and *a*_d_ with constant intervals (see Materials and Methods). For example, when *d* = 4 and *a*_4_ = 0, we set *a*_2_ and *a*_3_ to 0.666 and 0.333, respectively. This means that the QTL effects on the first to fourth variates become *B*_tQTL,1_, 0.666*B*_tQTL,1_, 0.333*B*_tQTL,1_, and 0, respectively. If we focus on variates 1 and 2 and examine the heat map for *d* = 2 (Figure 1), with this *a*_d_ value (0.666), the multivariate *F*-test is superior to the univariate test within the range from *r* = 0 to 0.3, which is consistent with the results when *d* = 4 (Figure 1). These results suggest that, although *a*_*j*_ will take various values when *d* > 2 in real-data analysis, the behavior of the multivariate *F*-test can be interpreted based on the results when *d* = 2 to some extent.

**Figure 3:**
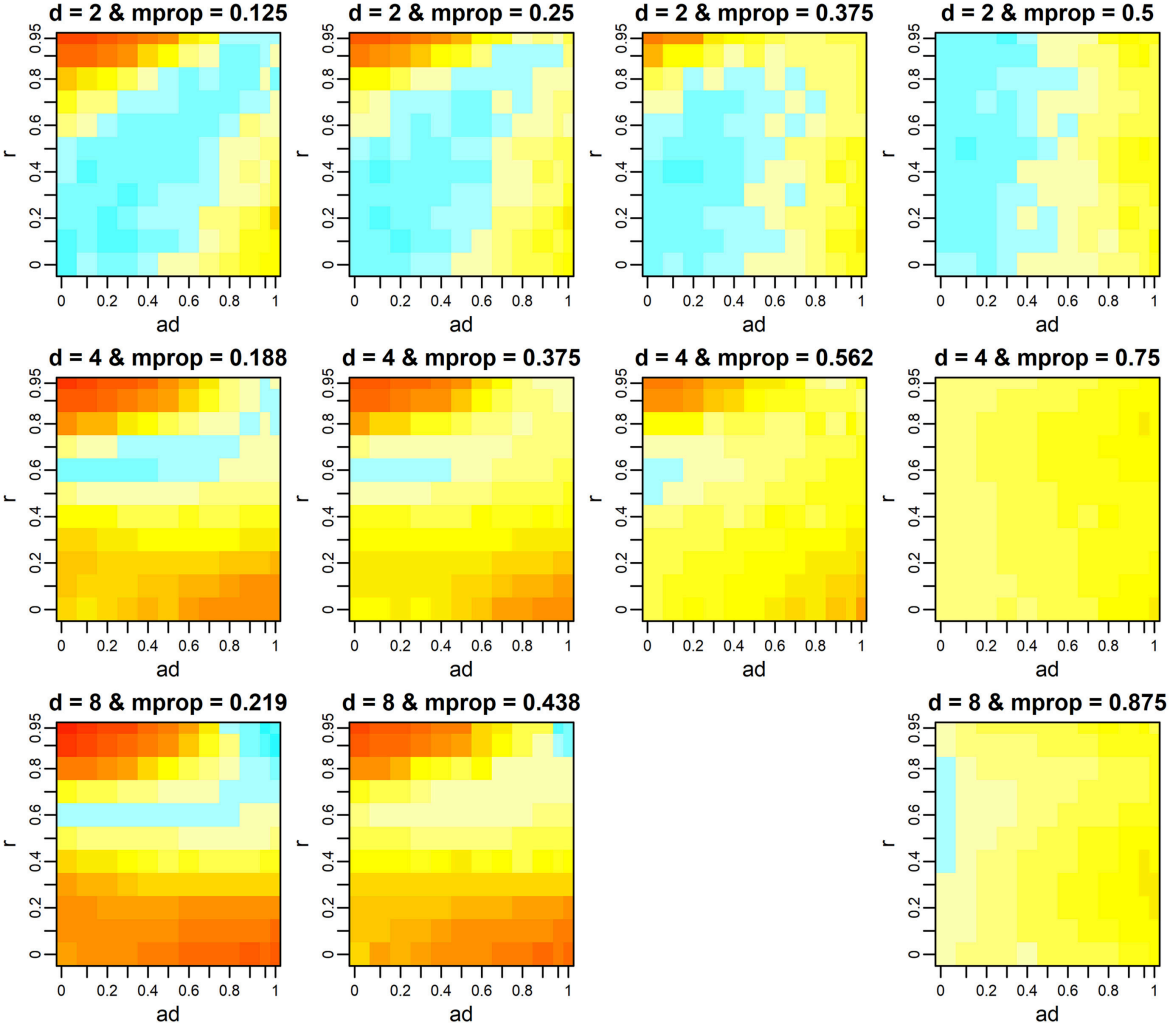
Mean difference of –log10*p*-values between the multivariate and univariate *F*-tests when phenotypes are missing. The *p*-values of the univariate *F*-test were obtained from the test on the first variate, which had the greatest QTL effect. Color scales are the same as those in Figure 1. *d, r*, and *a*_d_ denote the number of variates, phenotypic correlation, and the relative size of QTL effects between variates, respectively. *m*_prop_ denotes the missing proportion of phenotypes. At each *d*, the results when *m*_prop_ is the maximum (i.e., each individual has a phenotypic record only for a single variate) are presented in the last column. The results when *d* = 8 and *m*_prop_ = 0.656 (the third column on the bottom row) are not shown because of the high frequency of failure in variance component estimation. For results when *d* = 4 and *d* = 8, the mean differences at the scenarios that were not tested were interpolated using spline regression implemented in the mgcv R package.

For each *d*, as the missing proportion increased, the range of combinations of *r* and *a*_d_ where the multivariate *F*-test showed superiority gradually increased (Figure 3). This tendency was prominent when the number of variates was high. In contrast, the difference of–log10*p* (i.e., the surface of the heat map) became similar (flatter) among the simulation scenarios. Note that, from Figure 3, we dropped the results when *d* = 8 and *m*_prop_ = 0.656 because of a failure of variance component estimation in 98.1% of analyzes. We return to this issue later. When *m*_prop_ was the maximum, i.e., when every individual had a phenotypic value only for a single variate, the superiority of the multivariate *F*-test depended on *a*_d_, i.e., as *a*_d_ increased, the test outperformed the univariate one. However, it was also observed that the gain in–log10*p* was minimal. The fact that *r* became less influential when *m*_prop_ was the maximum can be illustrated analytically. Suppose that *d* = 2 (variates 1 and 2) and the first half of individuals (group X) has values only for variate 1 and the second half (group Y) has values only for variate 2. Let

$$\begin{array}{l} \Sigma_{u}^{2}=\left[\begin{array}{c} \sigma_{u1}^{2}\;r\sigma_{u1}\sigma_{u2}\\ r\sigma_{u1}\sigma_{u2}\;\sigma_{u2}^{2} \end{array}\right],\\ \Sigma_{e}^{2}=\left[\begin{array}{c} \sigma_{e1}^{2}\;r\sigma_{e1}\sigma_{e2}\\ r\sigma_{e1}\sigma_{e2}\;\sigma_{e2}^{2} \end{array}\right],and\\ A=\left[\begin{array}{c} A_{X}\;A_{XY}\\ A_{XY}\;A_{Y} \end{array}\right]. \end{array}$$

Then, the phenotype (co)variance matrix appearing in equation (2) can be written as

$$\begin{array}{l} V_{m}\;=\left[\begin{array}{c} \left[\begin{array}{c} A_{X}\;A_{XY}\\ A_{XY}\;A_{Y} \end{array}\right]\sigma_{u1}^{2}\;\left[\begin{array}{c} A_{X}\;A_{XY}\\ A_{XY}\;A_{Y} \end{array}\right]r\sigma_{u1}\sigma_{u2}\\ \left[\begin{array}{c} A_{X}\;A_{XY}\\ A_{XY}\;A_{Y} \end{array}\right]r\sigma_{u1}\sigma_{u2}\;\left[\begin{array}{c} A_{X}\;A_{XY}\\ A_{XY}\;A_{Y} \end{array}\right]\sigma_{u2}^{2} \end{array}\right]\\ +\left[\begin{array}{c} \left[\begin{array}{c} I_{X}\;0\\ 0\;I_{Y} \end{array}\right]\sigma_{e1}^{2}\;\left[\begin{array}{c} I_{X}\;0\\ 0\;I_{Y} \end{array}\right]r\sigma_{u1}\sigma_{u2}\\ \left[\begin{array}{c} I_{X}\;0\\ 0\;I_{Y} \end{array}\right]r\sigma_{u1}\sigma_{u2}\;\left[\begin{array}{c} I_{X}\;0\\ 0\;I_{Y} \end{array}\right]\sigma_{e2}^{2} \end{array}\right] \end{array}$$

where **I**_X_ and **I**_Y_ are the identity matrices for groups *X* and *Y*, respectively. Because groups *X* and *Y* do not have values for variates 2 and 1, respectively, the relevant rows and columns are removed from ***V***_m_ resulting in:

$$V_{m}=\left[\begin{array}{c} A_{X}\sigma_{u1}^{2}\;A_{XY}r\sigma_{u1}\sigma_{u2}\\ A_{XY}r\sigma_{u1}\sigma_{u2}\;A_{Y}\sigma_{u2}^{2} \end{array}\right]+\left[\begin{array}{c} I_{X}\sigma_{e1}^{2}\;0\\ 0\;I_{Y}\sigma_{e2}^{2} \end{array}\right].$$

The parameter *r* disappears from the residual covariance (the second term). Although *r* still appears in the genetic covariance (the first term), the influence of *r* on the *F*-value is expected to decrease.

To examine the multivariate *F*-test not inflating the *p*-values of negative SNPs, QQ plots were drawn for the SNPs that were on the same chromosome as the target QTLs and were not associated with any QTLs (i.e., *r*^2^ < 0.1 with any QTLs; Figures S2–S5). The results showed that the multivariate *F*-tests did not inflate the *p*-values of negative SNPs. Rather, *p*-values tended to be overestimated as *d* increased (Figures S4, S5).

A major drawback of the multivariate *F*-test is the computational difficulty in estimating the variance components, which became severe as *d* and *m*_prop_ increased. We observed that the solver we used (remlf90) occasionally failed to converge within the default iteration number (5,000) or to return a positive-definite matrix. As mentioned above, when *d* = 8 and *m*_prop_ = 0.656, the estimation failed in most cases in this scenario. In other scenarios, the frequencies of failure were 0.004, 0.054, and 0.081 when *d* = 2, 4, and 8, respectively. This issue would be solved using Gibbs sampling or by estimating the covariance for each pair of variates independently, as is often done in the genetic analysis of multiple traits (e.g., Nogi et al., 2011). When missing records are included, this issue may be mitigated by imputation. Computational speed also may be problematic, particularly when *n* and *d* increase. The most time-consuming step for evaluation of the *F*-value is the calculation of ${\text{X}}_{\text{m}}^{\prime }{\text{V}}_{\text{m}}^{-1}{\text{Y}}_{\text{m}}$, which is proportional to *n*(*n* + 1)*d*^3^. Considering this computational difficulty, meta-analyzes will be an attractive alternative to use multivariate information. For example, TETAS can combine *p*-values obtained by univariate mixed-effect models considering correlations between variates (van der Sluis et al., 2013). Comparing such meta-analysis methods with *F*-tests based on multivariate mixed-effects models is interesting, and we leave this issue for future studies.

As mentioned in the Materials and Methods, explicitly, the statistics in equations (1) and (2) do not follow the *F* distributions with the denominator degrees of freedom, *n* − *p* and *n* × *d* − *p*, respectively. However, we did not observe inflation of −log10*p*-values in simulations (Figures S2–S5), and thus we consider that the reduction of the denominator degrees of freedom owing to variance component estimation is not influential given the sample size in GWA. When accurate *p*-values are required, several remedies are available. The first one is permutation or parametric bootstrapping. These methods are time-consuming because, for each SNP, the null model is fitted to the generated data repeatedly to obtain the null distribution. Thus, genome-wide scans using these methods will not be feasible. Nevertheless, these methods will be useful for obtaining accurate *p*-values for SNPs that are pruned using other methods, including *F*-tests. The second one is to adjust the statistics such that the statistics follow *F* distributions as proposed in Kenward and Roger (1997). The method estimates the denominator degrees of freedom and a scaling parameter to match the moments of the statistics to those of the *F* distributions. Several extensions to multivariate models can be found in Alnosaier (2007). Although pursuing this second approach in GWA may be important from the theoretical viewpoint, in practice, applying permutation or parametric bootstrapping to a limited number of SNPs detected by *F*-tests or other methods would be useful.

When missing records are included, both the multivariate and univariate *F*-tests can be conducted for imputed data. Phenotype imputation is essential for several estimation algorithms for multivariate mixed-effect models because of transformation using eigen vectors, which is required for high computational efficiency (e.g., Zhou and Stephens, 2014; Lee and van der Werf, 2016). Phenotype imputation using multivariate information has been proposed for GWA (Dahl et al., 2016) and plant breeding (Hori et al., 2016). Although we evaluated the multivariate *F*-test without imputation in the present study, the performance of the multivariate and univariate *F*-tests based on imputed data would also depend on the magnitude of correlation between variates (*r*) as observed here because it will influence the imputation accuracy regardless of the imputation methods.

## Author Contributions

The author confirms being the sole contributor of this work and has approved it for publication.

### Conflict of Interest Statement

The author declares that the research was conducted in the absence of any commercial or financial relationships that could be construed as a potential conflict of interest.

## References

[B1] Alnosaier W. S. (2007). Kenward-Roger Approximate F Test for Fixed Effects in Mixed Linear Models. Doctoral Dissertation. Oregon State University.

[B2] Banerjee S. Yandell B. S. Yi N. (2008). Bayesian quantitative trait loci mapping for multiple traits. Genetics 179, 2275–2289. 10.1534/genetics.108.088427 18689903 PMC2516097

[B3] Cheng R. Doerge R. W. Borevitz J. (2017). Novel resampling improves statistical power for multiple-trait QTL mapping. G3 7, 813–822. 10.1534/g3.116.037531 28064191 PMC5345711

[B4] Dahl A. Iotchkova V. Baud A. Johansson A. Gyllensten U. Soranzo N.. (2016). A multiple-phenotype imputation method for genetic studies. Nat. Genet. 48, 466–472. 10.1038/ng.3513 26901065 PMC4817234

[B5] Endelman J. B. (2011). Ridge regression and other kernels for genomic selection with R package rrBLUP. Plant. Genome 4, 250–255. 10.3835/plantgenome2011.08.0024

[B6] Ferreira M. A. Purcell S. M. (2009). A multivariate test of association. Bioinformatics 25, 132–133. 10.1093/bioinformatics/btn563 19019849

[B7] Frischknecht M. Bapst B. Seefried F. R. Signer-Hasler H. Garrick D. Stricker C.. (2017). Genome-wide association studies of fertility and calving traits in Brown Swiss cattle using imputed whole-genome sequences. BMC Genomics 18:910. 10.1186/s12864-017-4308-z 29178833 PMC5702100

[B8] Furlotte N. A. Eskin E. (2015). Efficient multiple-trait association and estimation of genetic correlation using the matrix-variate linear mixed model. Genetics 200, 59–68. 10.1534/genetics.114.171447 25724382 PMC4423381

[B9] Galesloot T. E. van Steen K. Kiemeney L. A. L. M. Janss L. L. Vermeulen S. H. (2014). A comparison of multivariate genome-wide association methods. PLoS ONE 9:e95923. 10.1371/journal.pone.0095923 24763738 PMC3999149

[B10] Guo X. Li Y. Ding X. He M. Wang X. Zhang H. (2015). Association tests of multiple phenotypes: ATeMP. PLoS ONE 10:e0140348. 10.1371/journal.pone.0140348 26479245 PMC4610695

[B11] Henderson C. R. (1984). Applications of Linear Models in Animal Breeding. 3rd Edn. ed Schaeffer L. R. Guelph, ON: University of Guelph.

[B12] Hori T. Montcho D. Agbangla C. Ebana K. Futakuchi K. Iwata H. (2016). Multi-task Gaussian process for imputing missing data in multi-trait and multi-environment trials. Theor. Appl. Genet. 129, 2101–2115. 10.1007/s00122-016-2760-9 27540725

[B13] Joo J. W. Kang E. Y. Org E. Furlotte N. Parks B. Hormozdiari F.. (2016). Efficient and accurate multiple-phenotype regression method for high dimensional data considering population structure. Genetics 204, 1379–1390. 10.1534/genetics.116.189712 27770036 PMC5161272

[B14] Kang H. M. Sul J. H. Service S. K. Zaitlen N. A. Kong S. Y. Freimer N. B.. (2010). Variance component model to account for sample structure in genome-wide association studies. Nat. Genet. 42, 348–354. 10.1038/ng.548 20208533 PMC3092069

[B15] Kennedy B. W. Quinton M. van Arendonk J. A. (1992). Estimation of effects of single genes on quantitative traits. J. Anim. Sci. 70, 2000–2012. 10.2527/1992.7072000x 1644672

[B16] Kenward M. G. Roger J. H. (1997). Small sample inference for fixed effects from restricted maximum likelihood. Biometrics 53, 983–997. 10.2307/2533558 9333350

[B17] Kim S. Xing E. P. (2009). Statistical estimation of correlated genome associations to a quantitative trait network. PLoS Genet. 5:e1000587. 10.1371/journal.pgen.1000587 19680538 PMC2719086

[B18] Korte A. Vilhjalmsson B. J. Segura V. Platt A. Long Q. Nordborg M. (2012). A mixed-model approach for genome-wide association studies of correlated traits in structured populations. Nat. Genet. 44, 1066–1071. 10.1038/ng.2376 22902788 PMC3432668

[B19] Lee S. H. van der Werf J. H. (2016). MTG2: an efficient algorithm for multivariate linear mixed model analysis based on genomic information. Bioinformatics 32, 1420–1422. 10.1093/bioinformatics/btw012 26755623 PMC4848406

[B20] Liang L. Zollner S. Abecasis G. R. (2007). GENOME: a rapid coalescent-based whole genome simulator. Bioinformatics 23, 1565–1567. 10.1093/bioinformatics/btm138 17459963

[B21] Minamikawa M. F. Nonaka K. Kaminuma E. Kajiya-Kanegae H. Onogi A. Goto S.. (2017). Genome-wide association study and genomic prediction in citrus: potential of genomics-assisted breeding for fruit quality traits. Sci. Rep. 7:4721. 10.1038/s41598-017-05100-x 28680114 PMC5498537

[B22] Misztal I. Tsuruta S. Strabel T. Auvray B. Druet T. Lee D. H. (2002). BLUPF90 and related programs, in Proc. 7th World Congr. Genet. Appl. Livest. Prod. (Montpellier).

[B23] Nogi T. Honda T. Mukai F. Okagaki T. Oyama K. (2011). Heritabilities and genetic correlations of fatty acid compositions in longissimus muscle lipid with carcass traits in Japanese Black cattle. J. Anim. Sci. 89, 615–621. 10.2527/jas.2009-2300 21036930

[B24] O'Reilly P. F. Hoggart C. J. Pomyen Y. Calboli F. C. Elliott P. Jarvelin M. R.. (2012). MultiPhen: joint model of multiple phenotypes can increase discovery in GWAS. PLoS ONE 7:e34861. 10.1371/journal.pone.0034861 22567092 PMC3342314

[B25] Piepho H. P. (2005). Statistical tests for QTL and QTL-by-environment effects in segregating populations derived from line crosses. Theor. Appl. Genet. 110, 561–566. 10.1007/s00122-004-1872-9 15655665

[B26] R Development Core Team (2011). R: A Language and Environment for Statistical Computing. Vienna: R Foundation for Statistical Computing.

[B27] Ray D. Pankow J. S. Basu S. (2016). USAT: a unified score-based association test for multiple phenotype-genotype analysis. Genet. Epidemiol. 40, 20–34. 10.1002/gepi.21937 26638693 PMC4785800

[B28] Sahana G. Guldbrandtsen B. Thomsen B. Holm L. E. Panitz F. Brondum R. F.. (2014). Genome-wide association study using high-density single nucleotide polymorphism arrays and whole-genome sequences for clinical mastitis traits in dairy cattle. J. Dairy. Sci. 97, 7258–7275. 10.3168/jds.2014-8141 25151887

[B29] van der Sluis S. Posthuma D. Dolan C. V. (2013). TATES: efficient multivariate genotype-phenotype analysis for genome-wide association studies. PLoS Genet. 9:e1003235. 10.1371/journal.pgen.1003235 23359524 PMC3554627

[B30] VanRaden P. M. (2008). Efficient methods to compute genomic predictions. J. Dairy. Sci. 91, 4414–4423. 10.3168/jds.2007-0980 18946147

[B31] Wang Z. Wang X. Sha Q. Zhang S. (2016). Joint analysis of multiple traits in rare variant association studies. Ann. Hum. Genet. 80, 162–171. 10.1111/ahg.12149 26990300 PMC4836983

[B32] Yano K. Yamamoto E. Aya K. Takeuchi H. Lo P. C. Hu L.. (2016). Genome-wide association study using whole-genome sequencing rapidly identifies new genes influencing agronomic traits in rice. Nat. Genet. 48, 927–934. 10.1038/ng.3596 27322545

[B33] Yu J. Pressoir G. Briggs W. H. Vroh B. I. Yamasaki M. Doebley J. F.. (2006). A unified mixed-model method for association mapping that accounts for multiple levels of relatedness. Nat. Genet. 38, 203–208. 10.1038/ng1702 16380716

[B34] Zhang Y. Xu Z. Shen X. Pan W. (2014). Testing for association with multiple traits in generalized estimation equations, with application to neuroimaging data. Neuroimage 96, 309–325. 10.1016/j.neuroimage.2014.03.061 24704269 PMC4043944

[B35] Zhang Z. Ersoz E. Lai C. Q. Todhunter R. J. Tiwari H. K. Gore M. A.. (2010). Mixed linear model approach adapted for genome-wide association studies. Nat. Genet. 42, 355–360. 10.1038/ng.546 20208535 PMC2931336

[B36] Zhao K. Tung C. W. Eizenga G. C. Wright M. H. Ali M. L. Price A. H.. (2011). Genome-wide association mapping reveals a rich genetic architecture of complex traits in *Oryza sativa*. Nat. Commun. 2:467. 10.1038/ncomms1467 21915109 PMC3195253

[B37] Zhou X. Stephens M. (2014). Efficient multivariate linear mixed model algorithms for genome-wide association studies. Nat. Methods 11, 407–409. 10.1038/nmeth.2848 24531419 PMC4211878

