# Supplementary material for: Comparison of F-tests for Univariate and Multivariate Mixed-Effect Models in Genome-Wide Association Mapping
Source: Front Genet. 2019 Feb 4;10:30. doi: 10.3389/fgene.2019.00030 (PMC6369166; doi:10.3389/fgene.2019.00030)

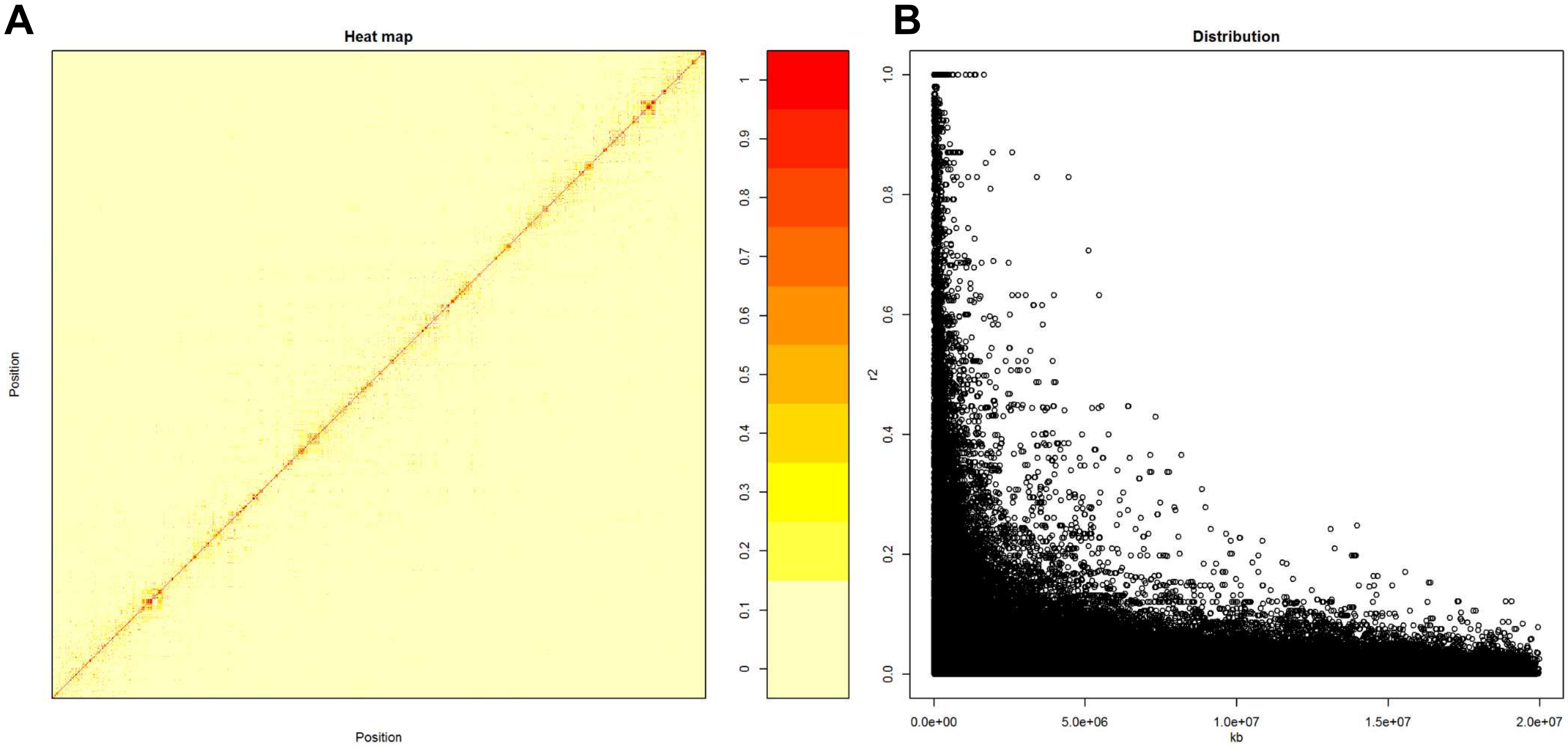

Figure S1. A typical distribution of linkage disequilibrium (r^2) between simulated SNPs. (A) Heat map of r^2. (B) r^2 across the physical distance. SNPs with minor allele frequency <0.02 were excluded.

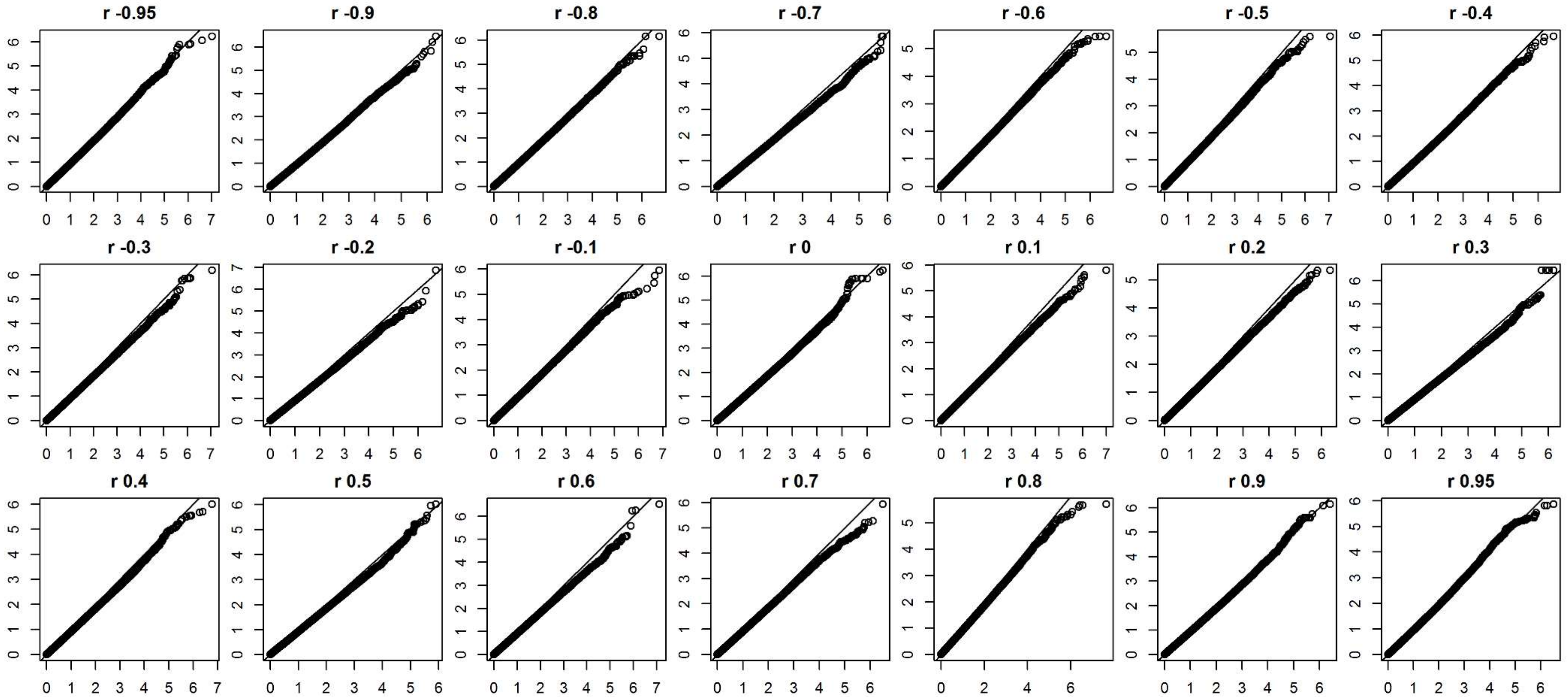

Figure S2. QQ plots for negative SNPs when d = 2 and m_{prop} = 0. The x-axis is the expected –log10p values, and the y-axis is the observed values. Negative SNPs are defined as the SNPs that are located at the same chromosome as the target QTLs and are not linked to any QTLs ($r^2 < 0.1$). P values were pooled across the parameter a_d (the relative size of QTL effects between variates). r in the figures indicates the phenotypic correlation.

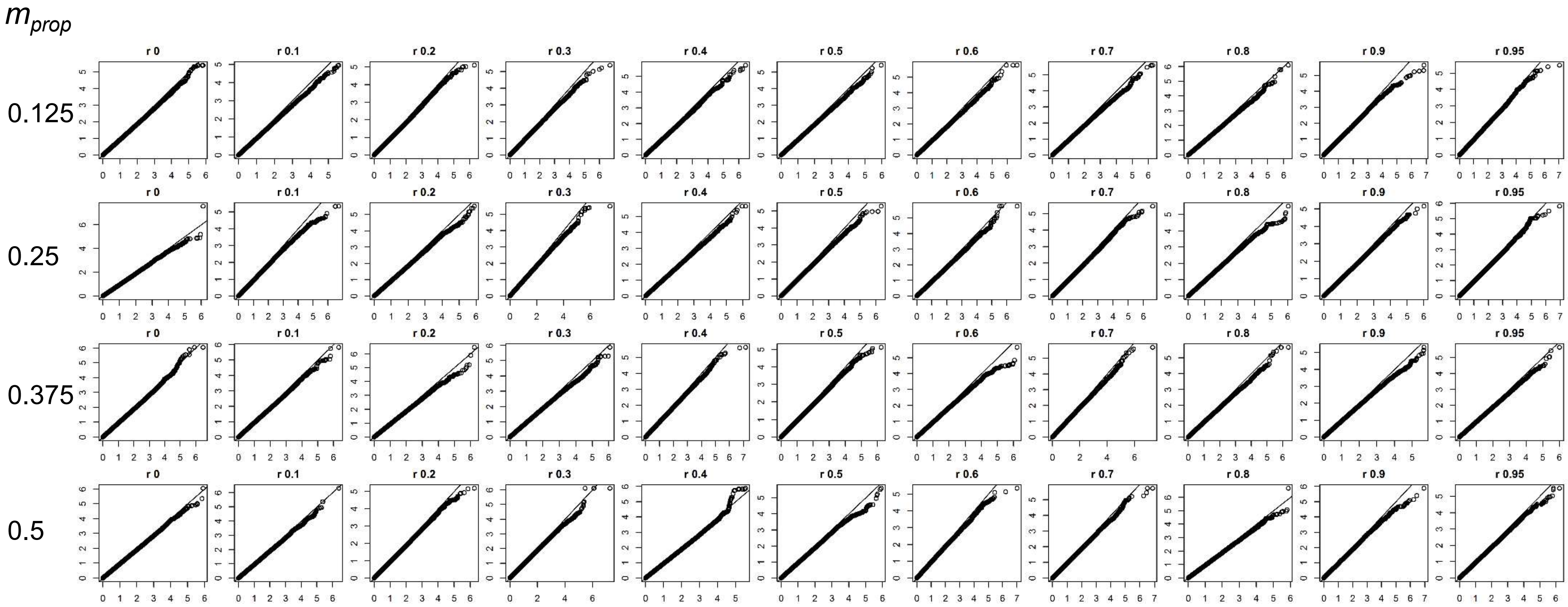

Figure S3. QQ plots for negative SNPs when d = 2 and m_{prop} > 0. The *x*-axis is the expected –log10*p* values, and the *y*-axis is the observed values. Negative SNPs are defined as the SNPs that are located at the same chromosome as the target QTLs and are not linked with any QTLs (r^2 < 0.1). *P* values were pooled across the parameter a_d (the relative size of QTL effects between variates). *r* in the figures indicates the phenotypic correlation.

Figure S4. QQ plots for negative SNPs when d = 4. The x-axis is the expected –log10p values, and the y-axis is the observed values. Negative SNPs are defined as the SNPs that are located at the same chromosome as the target QTLs and are not linked with any QTLs ($r^2 < 0.1$). P values were pooled across the parameter a_d (the relative size of QTL effects between variates). r in the figures indicates the phenotypic correlation.

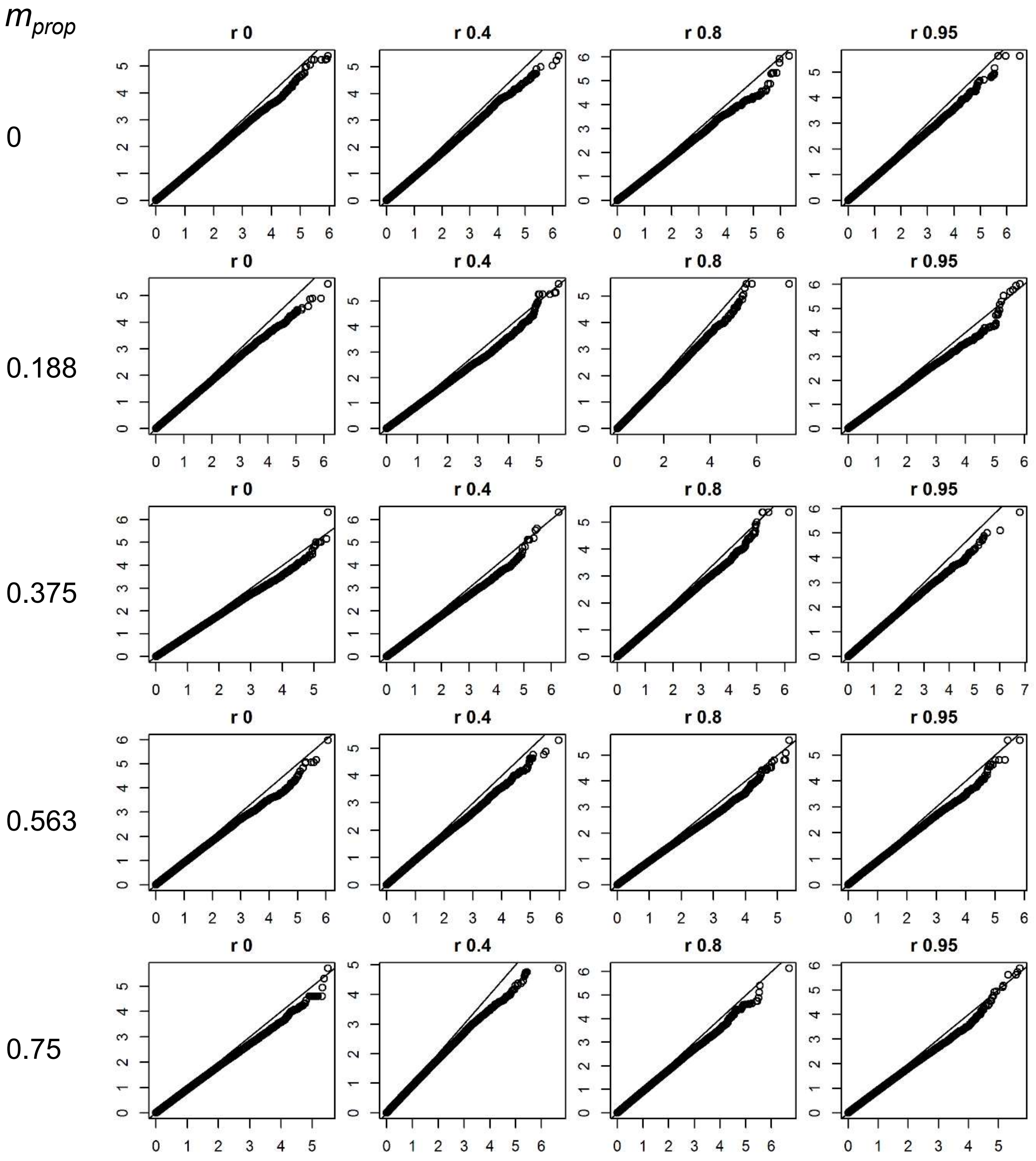

Figure S5. QQ plots for negative SNPs when d = 8. The x-axis is the expected –log10p values, and the y-axis is the observed values. Negative SNPs are defined as the SNPs that are located at the same chromosome as the target QTLs and are not linked with any QTLs ($r^2 < 0.1$). P values were pooled across the parameter a_d (the relative size of QTL effects between variates). r in the figures indicates the phenotypic correlation.

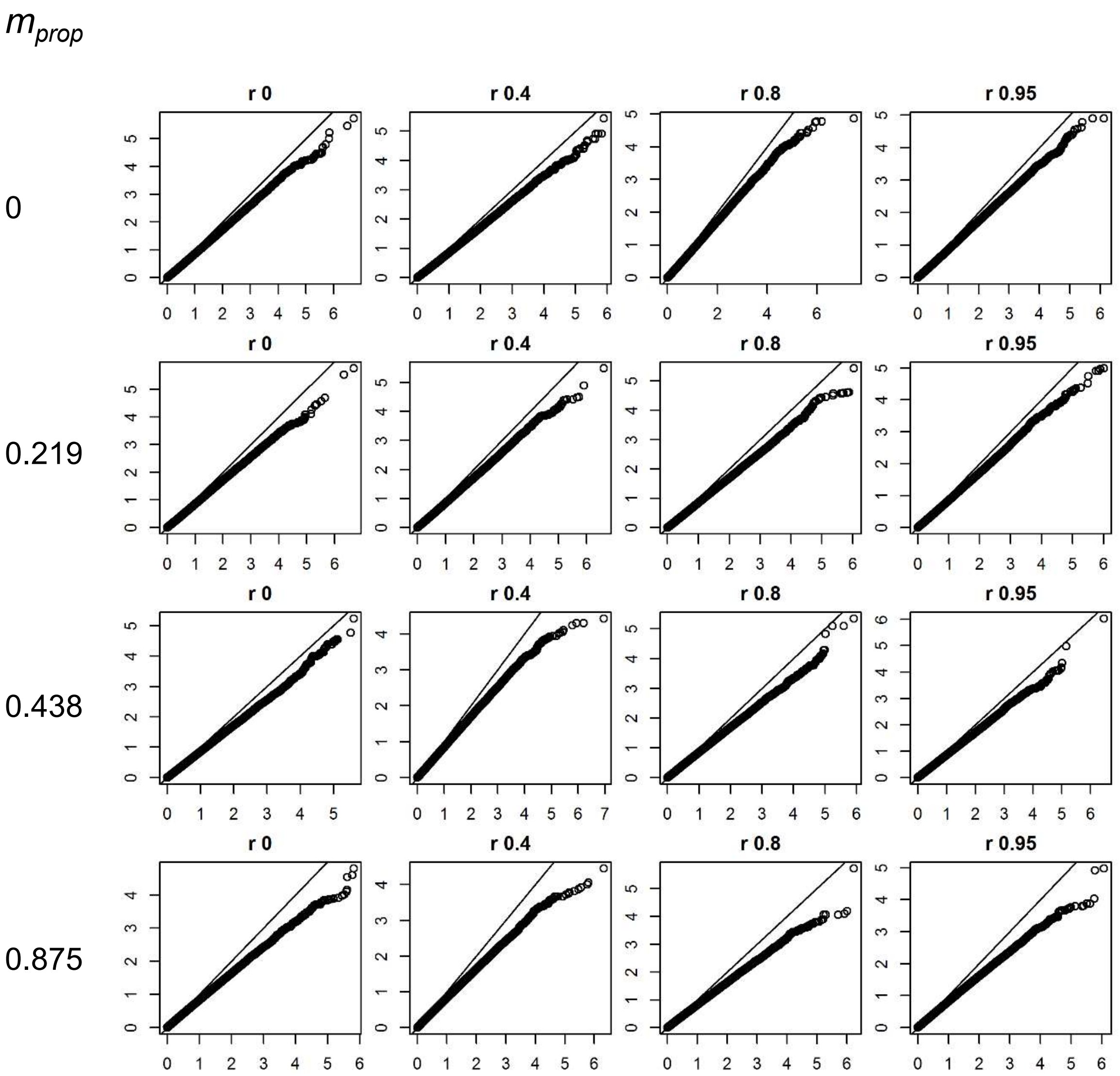

Supplement: Supplementary file 1 [file Data_Sheet_1.PDF]
